# Polymorphism in the tumour necrosis factor receptor II gene is associated with circulating levels of soluble tumour necrosis factor receptors in rheumatoid arthritis

**DOI:** 10.1186/ar1816

**Published:** 2005-09-07

**Authors:** John R Glossop, Peter T Dawes, Nicola B Nixon, Derek L Mattey

**Affiliations:** 1 Staffordshire Rheumatology Centre, University Hospital of North Staffordshire, Stoke-on-Trent, UK

## Abstract

Levels of soluble tumour necrosis factor receptors (sTNFRs) are elevated in the circulation of patients with rheumatoid arthritis (RA). Although these receptors can act as natural inhibitors of tumour necrosis factor-α, levels of sTNFRs in RA appear to be insufficient to prevent tumour necrosis factor-α induced inflammation. The factors that regulate circulating levels of sTNFRs are unclear, but polymorphisms in the tumour necrosis factor receptor genes may play a role. We investigated the relationship between polymorphisms in the tumour necrosis factor receptor I (TNF-RI) and II (TNF-RII) genes and levels of sTNFRs in two groups of Caucasian RA patients: one with early (disease duration ≤2 years; *n *= 103) and one with established disease (disease duration ≥5 years; *n *= 151). PCR restriction fragment length polymorphism analysis was used to genotype patients for the A36G polymorphism in the TNF-RI gene and the T676G polymorphism in TNF-RII. Levels of sTNFRs were measured using ELISA. We also isolated T cells from peripheral blood of 58 patients with established RA with known TNF-R genotypes, and release of sTNFRs into the culture medium was measured in cells incubated with or without phytohaemagglutinin. Serum levels of the two sTNFRs (sTNF-RI and sTNF-RII) were positively correlated in both populations, and the level of each sTNFR was significantly higher in the patients with established disease (*P *< 0.0001). Multiple regression analyses corrected for age, sex and disease duration revealed a significant trend toward decreasing sTNF-RI and sTNF-RII levels across the TNF-RII genotypes (TT > TG > GG) of patients with established disease (*P *for trend = 0.01 and *P *for trend = 0.03, respectively). A similar nonsignificant trend was seen for early disease. No relationship with the TNF-RI A36G polymorphism was observed. sTNFRs released by isolated T cells exhibited a similar trend toward decreasing levels according to TNF-RII genotype, although only the association with levels of sTNF-RII was significant. Strong correlations were found between levels of circulating sTNFRs and levels released by T cells *in vitro*. Our data indicate that the T676G polymorphism in TNF-RII is associated with levels of sTNFRs released from peripheral blood T cells, and with circulating levels of sTNFR in patients with RA.

## Introduction

Tumour necrosis factor (TNF)-α is a pleiotropic cytokine that is important in the pathogenesis of rheumatoid arthritis (RA), in which it plays a role in cartilage degradation, bone resorption, adhesion molecule expression, leucocyte infiltration, enzyme production and cytokine synthesis (see reviews by Brennan and coworkers [1] and Choy and Panayi [2]). The actions of TNF-α are mediated through binding to two distinct cell surface receptors, namely tumour necrosis factor receptor I (TNF-RI) and II (TNF-RII) [3,4]. Both are transmembrane glycoproteins with a three domain structure: a multiple cysteine-rich motif bearing an extracellular domain that facilitates ligand binding; a hydrophobic membrane spanning domain; and an intracellular domain that mediates signal transduction. The receptor molecules share significant homology in their extracellular domains but they have distinct intracellular domains [5]. Most significantly, TNF-RI, but not TNF-RII, possesses a death domain that can transduce the signal for cell death [6]. The two receptors appear to promote distinct TNF-α-induced cellular responses, although both are capable of inducing the nuclear factor-κB (NF-κB) and apoptotic pathways [7-10], providing some evidence of receptor function redundancy.

In addition to membrane bound forms, both TNF receptors can exist as soluble proteins. These are soluble variants of the extracellular domains [11-13] and are derived from the membrane bound form by the proteolytic actions of a disintegrin metalloproteinase called TNF-α converting enzyme (TACE) [14]. They retain their ligand binding capacity after cleavage [11,13] and can act as natural inhibitors of TNF-α by sequestering soluble TNF-α and preventing it from binding to membrane-bound TNF receptor. The levels of soluble TNF receptors (sTNFRs) are elevated in the serum and synovial fluid of RA patients [15-17], but these levels appear to be insufficient to prevent the chronic inflammation promoted by TNF-α [16]. Furthermore, the expression of membrane-bound TNF receptor is increased on a variety of cells in RA synovium [18,19], facilitating prolonged TNF-α signalling and the continuation of TNF-α regulated processes. The factors that regulate the levels of sTNFR are unclear, but polymorphisms within the TNF receptor genes may play a role.

The genes encoding TNF-RI and TNF-RII have been mapped to chromosomes 12p13 and 1p36, respectively [20]. Numerous polymorphisms are present in these genes [21-23] and some have been investigated for their association with RA [24-30]. An association has been reported between a single nucleotide polymorphism (SNP) in exon 6 (T676G) of the TNF-RII gene and susceptibility to familial but not sporadic RA [24,25]. Two studies in sporadic RA showed no association between the T676G polymorphism in the TNF-RII gene and RA severity [27,29], although one report has suggested an association with functional severity [28]. The A36G polymorphism in exon 1 of the TNF-RI gene has been associated with a protective role in familial RA [30].

In this study we report that polymorphism in the TNF-RII gene, but not the TNF-RI gene, is associated with circulating levels of TNF receptors in a population of Caucasian RA patients, and that this polymorphism is also associated with levels of sTNFRs released *in vitro *by isolated T cells from RA patients.

## Materials and methods

### Patients

Two groups of Caucasian RA patients were studied. The first group had early disease (duration ≤2 years; *n *= 103) and the second had established disease (duration ≥5 years; *n *= 151; Table 1). The patients were all of British origin and resident in North Staffordshire, England, and satisfied the 1987 American College of Rheumatology criteria for RA [31]. All patients were receiving anti-inflammatory and/or antirheumatic therapy, with the majority of patients with established disease (>90%) being treated with one or more disease-modifying antirheumatic drugs. Steroids and cytotoxic drugs such as azathioprine or cyclophosphamide were being received by a small minority of individuals (<5%). No patients were being treated with anti-TNF-α agents. Radiographic damage was measured by scoring radiographs of the hands and feet using the method proposed by Larsen and coworkers [32], and functional outcome was assessed using the Health Assessment Questionnaire (HAQ) [33]. In patients with early RA, HAQ measurements were taken at recruitment into the study and at 5 years of follow up.

Serum, separated from clotted peripheral blood (5 ml) from each patient, was stored at -70°C until required for measurement of sTNFRs. Synovial fluid was also collected from 45 patients who presented with knee effusions at the time of blood collection. Fluids were centrifuged and separated from the resulting cell pellet, before storage at -70°C. The study was approved by the North Staffordshire local research ethics committee.

### Cell isolation and culture

T cells were isolated from fresh peripheral blood samples (4 ml) from RA patients with established disease (*n *= 58). Cell isolation was by negative selection using a modified density gradient centrifugation technique that utilizes novel tetrameric antibody complexes (RosetteSep; Stemcell Technologies Inc., Vancouver, Canada). Isolated T cells (2 × 10^5 ^cells/200 μl) were cultured in RPMI 1640 synthetic culture medium supplemented with 10% heat-inactivated foetal bovine serum, 100 units/ml penicillin, 100 μg/ml streptomycin and 10% autologous serum, in 96-well cell culture plates. Cultures were incubated, with or without phytohaemagglutinin (PHA; 10 μg/ml), at 37°C in a 5% carbon dioxide humidified air environment for 48 hours. Cell supernatants were then harvested and stored at -20°C until required for analysis of sTNFR levels.

### Genomic DNA isolation

Peripheral blood samples (4 ml) collected in EDTA tubes were obtained from each patient and were stored at -20°C. After thawing at 37°C, the genomic DNA was isolated using a DNAce MegaBlood Kit procedure as directed by the manufacturer (Bioline, London, UK).

### PCR primers

The following primer sequences were used to amplify a 183 base pair fragment containing the SNP at nucleotide 36 in exon 1 of the TNF-RI gene [21]: forward 5'-GAG CCC AAA TGG GGG AGT GAG AGG-3', and reverse 5'-ACC AGG CCC GGG CAG GAG AG-3'.

A 242 base pair fragment containing the SNP at nucleotide 676 in exon 6 of the TNF-RII gene was amplified with the following primer sequences [34]: forward 5'-ACT CTC CTA TCC TGC CTG CT-3'; and reverse 5'-TTC TGG AGT TGG CTG CGT GT-3'.

### PCR amplification and single nucleotide polymorphism genotyping

The fragment of interest from each of the TNF receptor genes was amplified using an identical reaction mixture and conditions that were described previously [27]. All amplification reactions were performed in a Flexigene Thermal Cycler unit (Techne [Cambridge] Limited, Cambridge, UK) using a 96-well, full-skirt heating block. During amplification wells were capped with PCR cap strips. Following amplification the products were stored at 4°C until required for genotyping by restriction fragment length polymorphism analysis [27].

### ELISA

Serum, synovial fluid and T cell supernatant levels of sTNF-RI and sTNF-RII were quantified using the respective Duoset ELISA Development Kit as directed by the manufacturer (R&D Systems Europe, Abingdon, UK). For determination of sTNF-RI levels, sera, synovial fluids and T-cell supernatants were diluted 1:10, 1:50 and 1:3, respectively. For soluble TNF-RII, sera, synovial fluids and T-cell supernatants were diluted 1:20, 1:80 and 1:4, respectively. All samples were run in duplicate with the appropriate standards on 96-well microplates.

### Statistical analysis

The relationship between the two sTNFRs was assessed using Spearman's rank correlation, whereas differences in sTNFR levels between early and established RA were assessed using the Mann–Whitney U-Test. Multiple regression analysis was used to assess the relationship between each sTNFR and age (corrected for sex and disease duration), and between the TNF receptor genotypes and sTNFR levels (corrected for age, sex and disease duration). Where necessary the data were normalized by logarithmic transformation before analysis. All data were analyzed using the Number Cruncher Statistical Software package for Windows (NCSS 2000, NCSS Statistical Software, Kaysville, Utah, USA) *P *< 0.05 were considered statistically significant.

## Results

### sTNFR levels in rheumatoid arthritis

Both sTNFRs were detected in the sera of all patients studied. Consistent with the findings reported by Cope and coworkers [16], levels of sTNF-RII were approximately three times greater on average than those of sTNF-RI. A strong positive correlation was observed between the levels of the two sTNFRs in both patient populations (R_s _> 0.45; *P *< 0.0001). The levels of each sTNFR were also found to increase significantly with age in both patient groups (*P *< 0.0001), and this was independent of disease duration. Similar associations between sTNFR levels and age were seen in male and female patients (data not shown). Also, the median level of each sTNFR was significantly higher in patients with established disease than in those with early disease (*P *< 0.0001); this association remained after correction for age.

### TNF-RI A36G single nucleotide polymorphism and sTNFR serum levels

The A36G SNP genotype frequencies in each population and the respective mean levels of both sTNFRs are shown in Table 2. The observed allele frequencies for the A and G alleles were 64.1% and 35.9%, respectively, in the early RA population and 55.0% and 45.0% in the established RA population. The allele and genotype frequencies are broadly comparable to those reported elsewhere [24,27,30], although there is a suggestion from this study that the GG genotype is more frequent in patients with established disease. There were no significant differences in the serum levels of either sTNFR between the three genotypes in patients with early disease or with established disease (Table 2). This finding was also observed when the two populations were combined and the analysis repeated (data not shown).

### TNF-RII T676G single nucleotide polymorphism and sTNFR serum levels

The genotype and sTNFR level data for the T676G polymorphism in patients with early and established disease are shown in Table 3. The T and G alleles had frequencies of 77.2% and 22.8%, respectively, in both the early and established disease populations, and these frequencies were similar to those previously reported [24-29]. In established RA, analysis by multiple regression with correction for age, sex and disease duration revealed a significant association between TNF-RII genotype and the levels of sTNF-RI (*P *for trend = 0.01) and sTNF-RII (*P *for trend = 0.03) in the order TT > TG > GG. An identical trend was seen for levels of sTNF-RI and sTNF-RII in patients with early disease, although these associations were not significant (*P *= 0.3 and *P *= 0.055, respectively). In addition, the levels of sTNF-RI and sTNF-RII were significantly associated with TNF-RII genotype (*P *for trend = 0.02 and *P *for trend = 0.01, respectively) when the two populations were combined and analyzed by multiple regression with correction for age, sex and disease duration.

### TNF receptor polymorphisms and sTNFR synovial fluid levels

Synovial fluids collected at the same time as sera were available in 45 patients. Mean levels of sTNF-RI and sTNF-RII in the synovial fluids were significantly higher (7,736 and 18,120 pg/ml, respectively) than in the paired sera, but there was no direct correlation between levels in the synovial fluid and serum. No association was found between synovial fluid sTNFR levels and the A36G TNF-RI or 676G TNF-RII genotypes (data not shown).

### TNF receptor polymorphisms and clinical outcome measures

We showed previously that polymorphisms in the TNF-RI and TNF-RII genes were not associated with radiographic or functional severity in a cross-sectional study of patients with RA [27]. Similar findings were later reported by van der Helm-van Mil and coworkers [29], although another study by Constantin and colleagues [28] suggested an association of the TNF-RII G allele with worse functional (HAQ) outcome in early RA patients followed up for 5 years.

In the present study we again found no association between TNF-RI or TNF-RII polymorphisms and cross-sectional measures of radiographic or functional severity in patients with early or established disease (data not shown). In a similar manner to that reported by Constantin and coworkers [28], we also investigated the association between the TNF-RII polymorphism and functional severity of the early RA patients examined at baseline and at 5 years follow up. There was no significant difference in HAQ scores between patients with and those without the G allele at baseline (1.41 versus 1.60; *P *= 0.1) or after 5 years of follow up (1.41 versus 1.50; *P *= 0.9). There was also no significant difference in HAQ score progression.

Analysis of other clinical parameters associated with disease severity (extra-articular disease/nodules, rheumatoid factor, surgery, mechanical joint score, etc.) revealed no differences between TNF-RII genotypes (data not shown). However, in a separate study on anaemia in RA, involving many of these patients, we reported an association between carriage of the TNF-RII T allele and anaemia of chronic disease [35].

### TNF receptor polymorphisms and levels of sTNFR released by isolated T cells

We investigated whether there was any association between polymorphism in the TNF receptor genes and levels of sTNFRs released into the culture medium of unstimulated and stimulated T cells from RA patients. No association was found between the TNF-RI A36G polymorphism and levels of sTNFRs released (data not shown). However, a significant trend was found in levels of sTNF-RII released into culture medium by both unstimulated and stimulated T cells according to the TNF-RII genotype in the order TT > TG > GG (unstimulated and stimulated, respectively:*P *for trend = 0.049 and *P *for trend = 0.02; Table 4). Similar trends for release of sTNF-RI were seen in unstimulated and stimulated T cells, although these did not achieve statistical significance.

### Relationship between circulating levels of sTNFR and *in vitro *release from T cells

We examined whether the levels of sTNFRs in the circulation of RA patients were reflected in the levels of sTNFRs released by peripheral blood T cells *in vitro*. Strong correlations were found between serum levels of both sTNFRs and levels of these receptors released from isolated T cells (Table 5). The circulating levels of each sTNFR were strongly correlated with levels released by both unstimulated and stimulated T cells.

## Discussion

We investigated whether SNPs in the TNF receptor genes are associated with circulating levels of the naturally occurring soluble form of these receptor molecules in patients with early and established RA. We report evidence of an association between the T676G polymorphism in TNF-RII and serum levels of both sTNF-RI and sTNF-RII in patients with established disease, with a trend toward decreasing levels across the genotypes in the order TT > TG > GG. An identical trend was observed in patients with early disease, although the data failed to reach statistical significance. There was no evidence of any association between the TNF-RI A36G polymorphism and the levels of either sTNFR, in early or established RA.

No association was found between TNF receptor genotypes and synovial fluid levels of sTNFRs. This is probably not surprising because no correlation was found between synovial fluid and serum levels of sTNFRs, which is consistent with previous data [17]. We suggest that the high levels of sTNFRs seen in synovial fluids reflect the high degree of local inflammation, where genetic regulation of sTNFR levels by the TNF-RII gene is likely to have less impact than other factors in such an inflammatory environment. In contrast, the levels seen in the circulation are more likely to reflect genetic regulation of sTNFR levels, because any genetic influence is less likely to be overwhelmed by inflammatory factors.

Our finding of an association between the TNF-RII polymorphism and circulating levels of sTNFRs is reinforced by our *in vitro *studies, which show an identical trend in the release of sTNFRs, according to genotype, by isolated T cells from RA patients. We also demonstrated that the levels of sTNFR released by T cells *in vitro *are very closely correlated with the levels of circulating sTNFRs in these patients. Release of sTNFRs was greatest in T-cell cultures from patients carrying the TNF-RII TT genotype. The same trend was seen both in unstimulated and PHA stimulated cells, although the association with TNF-RII genotype was significant only for levels of sTNF-RII.

We also measured sTNFR release by isolated monocytes *in vitro *and found a similar relationship between sTNFR levels and TNF-RII genotype in cells stimulated with or without lipopolysaccharide. However, this did not reach significance, and the correlation between TNF receptor levels released by monocytes and serum levels was weaker than for T cells (unpublished observations). In multiple regression analyses of serum TNF receptor levels, which included levels released by T cells and monocytes as independent variables, we found that only levels released by T cells were associated with serum levels (unpublished observations).

The association of the TNF-RII T676G polymorphism with circulating sTNF-RII levels is consistent with a previous study [36] that demonstrated higher levels of sTNF-RII in healthy individuals carrying a T allele. However, the association with sTNF-RI levels was unexpected because the two TNF receptor genes are encoded on separate chromosomes. It is not clear how polymorphism within the TNF-RII gene might influence levels of sTNF-RI, although the strong correlation between the levels of these soluble receptors indicates that their production and/or release are closely linked.

The T676G polymorphism in exon 6 of the TNF-RII gene occurs within the fourth cysteine-rich domain of the extracellular domain, close to a point where the proteolytic cleavage site for TACE is thought to lie [37]. The polymorphism results in a nonconservative amino acid substitution in which arginine, with a highly basic side chain, replaces methionine, which has a nonpolar side chain (methionine → arginine, M196R). The location and nature of this polymorphism suggests the possibility that processing of membrane bound TNF-RII by TACE might be affected. However, functional analysis of this polymorphism in TNF-RII transfected HeLa cells revealed no effects on the release of soluble receptors from the cell surface, nor any effect on physical binding parameters [38]. In contrast, our findings suggest that this polymorphism may play a role in the regulation of soluble receptor release in T cells. However, the possibility that the association is with another polymorphism in linkage disequilibrium cannot be ruled out.

Recently, the TNF-RII 196R variant was shown to have a significantly lower ability to induce direct NF-κB signalling via TNF-RII and to enhance TNF-RI dependent TNF-α induced apoptosis [39]. The diminished ability of the 196R variant to induce NF-κB activation is paralleled by a diminished induction of NF-κB dependent target genes involved in antiapoptotic or proinflammatory functions. It is possible that in certain cell types or under particular experimental conditions that the reduced ability of the 196R variant to induce NF-κB dependent genes may lead to reduced release of sTNFRs (e.g. through lower production of proteins that are important in regulating the cleavage and release of these receptors).

The mean serum levels of sTNF-RII were approximately three times greater than for sTNF-RI in each patient population. The levels of sTNF-RII in culture medium from unstimulated T cells were also about three times greater than those of sTNF-RI, although this increased to approximately five times greater after stimulation of T cells with PHA. This can be explained by increased release of sTNF-RII, but not of sTNF-RI, after PHA stimulation. This is similar to the situation previously observed in lipopolysaccharide stimulated monocytes and alveolar macrophages, in which sTNF-RII but not sTNF-RI release was enhanced after stimulation [40,41]. These differences in soluble receptor release may have important consequences for TNF-α signalling (e.g. increased release of sTNF-RII may reduce the ability of cells to be activated by interactions with membrane bound TNF-α on surrounding cells) [42]. Localized differences in the concentrations of soluble receptors may also have significant effects on inhibition or promotion of TNF-α activity, depending on the tissue compartment and the level of TNF-α present.

Previous studies in healthy individuals reported an age associated significant increase in serum levels of both sTNFRs [43,44], whereas a study conducted in RA patients failed to identify any correlation between sTNFR levels and age [16]. In another study conducted in healthy individuals [45] the levels of sTNF-RII were lower in older (50–67 years) than in younger individuals (25–35 years). In both populations studied here, we found a highly significant association between increasing levels of both sTNFRs and age, which was independent of disease duration. An explanation for these conflicting data is not yet evident.

The clinical relevance of our findings is unclear at present, but it has been shown that familial susceptibility to RA is associated with the TNF-RII G allele and particularly the GG genotype [24,25], which was associated with the lowest sTNF-RII levels in our study. It is possible that lower levels of sTNFRs may contribute to the development of RA if a particular threshold of TNF-α activity is exceeded in genetically susceptible individuals. Genetic regulation of TNF receptor levels may also influence the long-term outcome of the disease and response to anti-TNF-α therapy. There is some evidence that individuals carrying the TNF-RII G allele exhibit poorer response to anti-TNF-α therapy [46]. Constantin and coworkers [28] also suggested that the G allele is associated with worse functional outcome, based on 5 years of follow up of early RA patients, although we did not find an association in a previous cross-sectional study [27] and could not confirm the findings of those investigators in the present study. Therefore, the association of the TNF-RII T676G polymorphism with functional severity is uncertain. However, we have provided recent evidence that the T allele (associated with higher sTNFR serum levels and increased release from T cells in the present study) may be associated with anaemia of chronic disease in RA [35]. Compared with nonanaemic patients, those with anaemia of chronic disease also have serum levels of sTNF-RI and sTNF-RII that are about 30% higher (*P *≤ 0.007), which is consistent with the T allele association.

Although the differences in sTNFR serum levels between TNF-RII genotypes are not large, it is noteworthy that exactly the same trend was seen throughout for serum levels in early and established RA, and for unstimulated and stimulated T-cell cultures. Several studies have shown that circulating sTNFR levels and/or polymorphisms in the TNF-RII gene are associated with heart failure, hypertension, obesity and insulin resistance, and differences in serum levels of a similar magnitude to those found in this study were shown to be clinically relevant in these conditions [47-52]. Our findings may thus be of particular importance in RA, in which there is evidence of increased risk for cardiovascular disease and metabolic syndrome abnormalities [53].

## Conclusion

Our results indicate that there is an association between the T676G SNP in the TNF-RII gene and levels of sTNFRs released by T cells of RA patients. This finding is reinforced by an association between this polymorphism and circulating levels of sTNFRs in established RA. Although various inflammatory factors may influence the release of TNF receptors, our data indicate that genetic regulation involving the TNF-RII gene may play some role in determining circulating levels in RA.

## Abbreviations

ELISA = enzyme-linked immunosorbent assay; HAQ = health assessment questionnaire; NF-κB = nuclear factor-κB; PCR = polymerase chain reaction; PHA = phytohaemagglutinin; RA = rheumatoid arthritis; SNP = single nucleotide polymorphism; sTNFR = soluble tumour necrosis factor receptor; TACE = TNF-α converting enzyme; TNF = tumour necrosis factor; TNF-RI = tumour necrosis factor receptor I; TNF-RII = tumour necrosis factor receptor II.

## Competing interests

The author(s) declare that they have no competing interests.

## Authors' contributions

JRG carried out genotyping, cell isolation, cell culture, and ELISA work, and wrote the first draft of the paper. PTD gave advice on patient selection, study design and interpretation of data. NBN carried out some genotyping and ELISA work. DLM conceived and oversaw the study, carried out statistical analyses and interpretation of data, and finalized the manuscript. The final manuscript was read and approved by all authors.

## Figures and Tables

**Table 1:** Characteristics of the two rheumatoid arthritis patient populations

Population	Early disease	Established disease
Number	103	151
Male/Female (*n*)	44/59	63/88
Age (years; mean ± SD)	55.0 ± 13.4	60.2 ± 11.1
Age at onset (years; mean ± SD)	54.3 ± 13.4	46.6 ± 12.1
Disease duration (years; mean ± SD)	0.7 ± 0.5	13.7 ± 6.3
Rheumatoid factor ever positive (%)	52/92 (56.5)	86/126 (68.3)
Nodule positive (%)	4/102 (3.9)	28/150 (18.7)

SD, standard deviation.

**Table 2:** TNF-RI A36G single nucleotide polymorphism genotype frequencies and sTNFR levels

Genotype	*n *(%)	sTNF-RI (pg/ml)	sTNF-RII (pg/ml)
Early RA			
AA	41 (39.8)	1,543 ± 597	4,435 ± 1,898
AG	50 (48.5)	1,426 ± 629	4,302 ± 1,672
GG	12 (11.7)	1,303 ± 447	4,566 ± 1,490
Established RA			
AA	48 (31.8)	1,827 ± 758	5,740 ± 1,942
AG	70 (46.4)	1,688 ± 674	5,475 ± 2,020
GG	33 (21.8)	1,757 ± 559	5,857 ± 2,393

Shown are tumor necrosis factor receptor I (TNF-RI) A36G single nucleotide polymorphism genotype frequencies and soluble tumor necrosis factor receptor (sTNFR) levels in rheumatoid arthritis (RA) patients with early (*n *= 103) and established (*n *= 151) disease. sTNFR levels are expressed as the mean ± standard deviation. No significant differences in sTNFR levels were found between any of the genotypes in either population. sTNF-RII, soluble tumor necrosis factor receptor II.

**Table 3:** TNF-RII T676G single nucleotide polymorphism genotype frequencies and sTNFR levels

Genotype	*n *(%)	sTNF-RI (pg/ml)	sTNF-RII (pg/ml)
Early RA			
TT	63 (61.2)	1,503 ± 704	4,690 ± 1,961
TG	33 (32.0)	1,451 ± 370	3,961 ± 1,242
GG	7 (6.8)	1,094 ± 240	3,648 ± 697
Established RA			
TT	91 (60.3)	1,816 ± 705	5,837 ± 2,219
TG	51 (33.7)	1,633 ± 642	5,375 ± 1,921
GG	9 (6.0)	1,700 ± 570	5,187 ± 1,066

Shown are tumour necrosis factor receptor II (TNF-RII) T676G single nucleotide polymorphism (SNP) genotype frequencies and soluble tumour necrosis factor receptor (sTNFR) levels in rheumatoid arthritis (RA) patients with early (*n *= 103) and established (*n *= 151) disease. sTNFR levels are expressed as the mean ± standard deviation. Multiple regression analyses of log transformed data corrected for age, sex and disease duration revealed a significant trend of decreasing soluble tumour necrosis factor receptor I (sTNF-RI) and sTNF-RII levels across the genotypes (order: TT > TG > GG) of patients with established disease (*P *for trend = 0.01 and *P *for trend = 0.03, respectively). A similar nonsignificant trend was seen for patients with early disease (*P *= 0.3 and *P *= 0.055, respectively).

**Table 4:** Association between TNF-RII T676G single nucleotide polymorphism genotype and sTNFR levels released by T cells

Genotype	*n *(%)	sTNF-RI (pg/ml)	sTNF-RII (pg/ml)
Unstimulated T cells			
TT	38 (65.5)	166.8 ± 57.8	582.2 ± 259.6
TG	15 (25.9)	144.1 ± 78.2	428.1 ± 222.3
GG	5 (8.6)	137.2 ± 68.9	398.8 ± 194.9
Stimulated T cells			
TT	38 (65.5)	178.0 ± 57.9	998.3 ± 355.6
TG	15 (25.9)	146.5 ± 75.5	769.5 ± 292.8
GG	5 (8.6)	141.6 ± 75.7	724.4 ± 167.3

Shown is the association between tumour necrosis factor receptor II (TNF-RII) T676G single nucleotide polymorphism genotype and soluble tumour necrosis factor (sTNFR) levels released by T cells isolated from rheumatoid arthritis (RA) patients (*n *= 58). sTNFR levels are expressed as mean ± standard deviation. Levels of sTNFR released into culture medium of isolated T cells exhibited a similar trend of decreasing levels of both receptors according to TNF-RII genotype (order: TT > TG > GG), although only the associations with sTNF-RII were significant (unstimulated and stimulated cells, respectively: *P *for trend = 0.049 and *P *for trend = 0.02; multiple regression analysis corrected for age, sex and disease duration). sTNF-RI, soluble tumor necrosis factor receptor I.

**Table 5:** Correlation between serum levels of sTNFR and levels released by isolated T cells

Serum levels	Unstimulated T cells		Stimulated T cells	
	
	sTNF-RI	sTNF-RII	sTNF-RI	sTNF-RII
sTNF-RI	0.883	0.818	0.858	0.692
sTNF-RII	0.865	0.923	0.837	0.763

Shown is the correlation between serum levels of soluble tumour necrosis factor receptor (sTNFR) and levels released by isolated T cells from rheumatoid arthritis (RA) patients (*n *= 58). Spearman correlation coefficients are shown. *P *< 0.0001 for all correlations. sTNF-RI, soluble tumor necrosis factor receptor I; sTNF-RII, soluble tumor necrosis factor receptor II.
